# Evolution of miRNA Tailing by 3′ Terminal Uridylyl Transferases in Metazoa

**DOI:** 10.1093/gbe/evx106

**Published:** 2017-06-14

**Authors:** Vengamanaidu Modepalli, Yehu Moran

**Affiliations:** * Department of Ecology, Evolution and Behavior, Alexander Silberman Institute of Life Sciences, Faculty of Science, The Hebrew University of Jerusalem, Jerusalem, Israel

**Keywords:** TUTase, RNA tailing, RNA modification, microRNA

## Abstract

In bilaterian animals the 3′ ends of microRNAs (miRNAs) are frequently modified by tailing and trimming. These modifications affect miRNA-mediated gene regulation by modulating miRNA stability. Here, we analyzed data from three nonbilaterian animals: two cnidarians (*Nematostella vectensis* and *Hydra magnipapillata*) and one poriferan (*Amphimedon queenslandica*). Our analysis revealed that nonbilaterian miRNAs frequently undergo modifications like the bilaterian counterparts: the majority are expressed as different length isoforms and frequent modifications of the 3′ end by mono U or mono A tailing are observed. Moreover, as the factors regulating miRNA modifications are largely uncharacterized in nonbilaterian animal phyla, in present study, we investigated the evolution of 3′ terminal uridylyl transferases (TUTases) that are known to involved in miRNA 3′ nontemplated modifications in Bilateria. Phylogenetic analysis on TUTases showed that TUTase1 and TUTase6 are a result of duplication in bilaterians and that TUTase7 and TUTase4 are the result of a vertebrate-specific duplication. We also find an unexpected number of Drosophila-specific gene duplications and domain losses in most of the investigated gene families. Overall, our findings shed new light on the evolutionary history of TUTases in Metazoa, as they reveal that this core set of enzymes already existed in the last common ancestor of all animals and was probably involved in modifying small RNAs in a similar fashion to its present activity in bilaterians.

## Introduction

MicroRNAs (miRNAs) are produced through a series of complex enzymatic processes prior to taking their mature form. In general, primary miRNA (pri-miRNAs) processing is initiated by cleaving the pri-miRNAs by an RNAse III enzyme called Drosha and released into cytoplasm as premiRNA (Lee et al. 2003). In the cytoplasm premiRNAs are processed by another RNAse III enzyme, Dicer, which cleaves the overhangs of the premiRNAs and released it as a double stranded miRNA (Lee et al. 2002), later it interacts with Argonaute (AGO) and guides RNA-induced silencing complexes (RISC) to target mRNAs (Peters and Meister 2007; Bartel 2009). Typically, miRNAs are produced at the length of 22 nucleotides (nt), however, high-throughput sequencing studies have found there is heterogeneous combination of miRNA isoforms produced in addition to the dominant mature miRNA sequences (Langenberger et al. 2009). miRNA modifications include isoforms with variations at 3′ and 5′ ends and nontemplated nucleotide additions at 3′ end of miRNA (Burroughs et al. 2010; Han et al. 2011; Mansur et al. 2016). These modifications can lead to delay in maturation of miRNAs and also influence their stability and function (Ameres et al. 2010; Han et al. 2011; Wyman et al. 2011; Heo et al. 2012; Ji and Chen 2012; Knouf et al. 2013; Thornton et al. 2014). 3′ modification is not unique to miRNAs as a number of studies performed in other populations of RNAs revealed the significance of posttranscriptional modifications in regulating RNA stability and function (Anantharaman et al. 2002; Wang and He 2014). Such modifications include RNA editing catalyzed by adenosine deaminases (ADARs) (Nishikura 2016), single nucleotide polymorphism (SNPs) (Duan et al. 2007), tailing catalyzed by terminal uridylyl transferases (TUTases) (Lim, Ha, et al. 2014; Thornton et al. 2014), and methylation of 3′ end by the methyltransferase HEN1 (Ji and Chen 2012).

RNA tailing is one of the most frequent posttranscriptional modifications (Norbury 2013). Poly (A) polymerases (PAPs) are known to add a poly (A) tail to mRNAs and the key function of this addition of nontemplated nucleotides is the protection of the mRNA from 3′ to 5′ exonucleases in cytoplasm (Yamashita et al. 2005; Schmidt and Norbury 2010; Schoenberg and Maquat 2012). Apart from poly (A) tail, 3′ end of RNA can also undergo modifications by TUTases that add a uridine tail (Kwak and Wickens 2007; Mullen and Marzluff 2008; Rissland and Norbury 2009; Norbury 2013). These TUTases are assumed to evolve from ancestral PAPs by switching their nucleotide preference to UTP (Lunde et al. 2012; Yates et al. 2012). TUTases are responsible for the addition of mono and poly uridylyl tails to different RNA populations in a wide range of organisms. For example, in the fission yeast *Schizosaccharomyces pombe* the TUTase-related Cid1 protein family regulates mRNA turnover by targeting specific mRNAs and promoting their decapping (Stevenson and Norbury 2006; Rissland and Norbury 2009). The uridylation of small RNA 3′ was first identified in plants as HEN1 SUPPRESSOR1 (HESO1) and UTP: RNA uridylyltransferase (URT1) were found to be involved in decaying of uriydylated mature small RNAs in *Arabidopsis* (Li et al. 2005)*.* In human, seven TUTases were identified to date and were demonstrated to carry specialized functions. For example, TUTase4 (also known as ZCCHC11) is involved in uridylation of histone mRNAs and promotes their decay (Schmidt et al. 2011). TUTase7 (also known as ZCCHC6) is involved in monouridylation of group II premiRNAs, a set of premiRNAs lacking 3′ 2nt overhangs, which is required for Dicer processing. The majority of the let7 miRNA family members and miR-105 are shorter and lack 3′ 2nt overhangs. In such cases, TUTases 7, 4, and 2 are involved in generating the 2-nt 3′ overhang through mono-uridylation and by that enable Dicer processing (Heo et al. 2012; Kim et al. 2015). TUTase7, TUTase4 and TUTase2 (also known as GLD2) are responsible together for terminal uridylation of miRNAs (Heo et al. 2012; Thornton et al. 2014). TUTase6 (also called U6 TUTase) is involved in modification of U6 snRNAs by 3′ uridylation, leading to shortening of the 3′ end by exonucleases (Trippe et al. 2006; Mullen and Marzluff 2008; Rissland and Norbury 2009). A mitochondrial PAP (known as PAPD1 or TUTase1) is responsible for adding poly(A) tails in mammalian mitochondrial mRNAs, a modification that regulates the translation and stability of these mRNAs (Chang and Tong 2012). In overall these studies show that while tailing by TUTases is important for controlling RNA stability, it has different consequences on different RNA pathways.

Even though TUTases are conserved throughout Metazoa, little is known about their evolution as they were studied almost exclusively in few bilaterian models such as mammals, *Drosophila melanogaster*, *Caenorhabditis elegans*, and zebrafish. However, as small RNAs are found in nonbilaterian animal clades such as Porifera (sponges) and Cnidaria (sea anemones, corals, hydroids, and jellyfish) (Grimson et al. 2008; Wheeler et al. 2009; Moran et al. 2017), it is possible that TUTases also modulate their stability and that this regulatory pathway is ancestral to all animals. This notion is corroborated by the fact that the stability of small RNAs in plants is also modulated by trimming and tailing in a similar fashion to bilaterian animals (Ji and Chen 2012; Zhai et al. 2013; Wang et al. 2016).

To assay whether miRNA modifications such as nontemplated addition and trimming occur in nonbilaterian animals, we performed an in-depth analysis of small RNA sequence data from Cnidaria (*Nematostella vectensis* and *Hydra magnipapillata*) and Porifera (*Amphimedon queenslandic*a). Additionally, we analyzed the phylogeny of TUTases family throughout Metazoa and their holozoan relatives to unravel the evolutionary history of these RNA modifying enzymes.

## Materials and Methods

### Detection of Homologs of RNA Modifier Enzymes

Using protein sequences from human we performed Blast search for TUTases protein sequences from all major groups of animals and their holozoan relatives including Choanoflagellata, Filasterea, Porifera, Placozoa, Cnidaria, Ctenophora, Deuterostomia, Lophotrochozoa, and Ecdysozoma. The list of species considered for TUTase homologs search are provided in supplementary file (supplementary table 1, Supplementary Material online). We carried out BLASTP searches using the National Center for Biotechnology Information (NCBI) (BLAST+ 2.5.0 Fri, 23 September 2016 17:00:00 EST) (https://blast.ncbi.nlm.nih.gov/Blast.cgi; last accessed May 4, 2017) on all available metazoan species (under the term Metazoa) as well as on Choanoflagellata and Capsaspora using *Homo sapiens* protein sequences as queries. In case we found no matches we carried out TBLASTN searches with the same query sequences, using the Transcriptome Shotgun Assembly (TSA) database of NCBI and restricted it to Cnidaria and Porifera. In addition, for nonbilaterian species we carried out species-specific searches through Compagen (http://compagen.zoologie.uni-kiel.de/index.html; last accessed May 4, 2017) (Hemmrich and Bosch 2008), Joint Genome Institute (http://genome.jgi.doe.gov/; last accessed May 4, 2017) (Grigoriev et al. 2012) and the *Mnemiopsis* Genome Project Portal (https://kona.nhgri.nih.gov/mnemiopsis/blast/; last accessed May 4, 2017) (Moreland et al. 2014) databases. Further, with the retrieved matching sequences we carried out reciprocal BLAST searches against *Homo sapiens* and *Danio rerio* protein sequences. The *E*-values, percentage identity and similarity of all TUTase homologs from the phylogenetic study are provided in supplementary table 2, Supplementary Material online. Retrieved proteins were analyzed to determine conserved domains using the CDD tool (v3.15 27 June 2016) (Marchler-Bauer et al. 2015) and PFAM tool (v30.0, July 1, 2016) (Finn et al. 2014).

### Animals

*Nematostella vectensis* polyps were grown in 16 ‰ artificial sea water at 18 °C in the dark and fed three times a week with freshly hatched *Artemia salina* nauplii. Induction of spawning was performed as previously described (Genikhovich and Technau 2009). Samples for RNA extraction were flash frozen in liquid nitrogen and stored at −80 °C until used.

### Phylogenetic Analysis

The protein sequences were aligned using MUSCLE (Edgar 2004) algorithm in the SeaView program (Gouy et al. 2010) and the trimming was performed using TrimAl (Capella-Gutierrez et al. 2009) to remove any low quality regions. In order to identify the appropriate model for Phylogenic reconstruction we used ProTest (Darriba et al. 2011). The maximum-likelihood (ML) phylogenetic trees were constructed using PhyML v3.0 (Guindon et al. 2010), we used a starting tree constructed by BioNJ (implemented in PhyML) and used the tree searching option combining the best results of both NNI and SPR. Statistical tree robustness was assessed in PhyML via 100 bootstrap replicates. MrBayes v3.2.1 was used to construct Bayesian tree, the run lasted 5,000,000 generations and every 100th generation was sampled (Altekar et al. 2004). We used one cold and three heated chains in each run. All runs reached a standard deviation of split frequencies of 0.0006 or lower. The first 25% of the samples were discarded by burn-in. In all runs minimal estimated sample size was calculated to be above 15,000, indicating adequate sampling, and a PSRF value of 1.0 was reached, which indicates convergence.

### Small RNA Library Preparation

Total RNA was extracted from different developmental stages of *Nematostella vectensis* (blastula, primary polyp, late planula, adult male, and female) using Tri-Reagent (Sigma-Aldrich) following the manufacturer’s instructions. Three biologically independent animal pools were used for each developmental stage. From each stage 20 µg of total RNA was used and the RNA integrity was analyzed with a Bioanalyzer (Agilent Technologies, USA). The small RNA size selection was performed using 15% denaturing urea polyacrylamide gel, RNA elution from gel was performed overnight and precipitated using ethanol. Small RNA libraries were constructed as described in Phillip Zamore Lab Illumina TruSeq Small RNA Cloning Protocol April, 2014 (http://www.umassmed.edu/zamore/resources/protocols/; last accessed May 4, 2017). Small RNA libraries were sequenced in the Center for Genomic Technologies of the Hebrew University on the NextSeq 500 platform (Illumina) with 50 nt read length. The raw data have been deposited at NCBI GEO Submission (GSE94526) (supplementary table 3, Supplementary Material online).

### Bioinformatics Analysis

The sequencing data were preprocessed to remove the adapters using Cutadapt (Martin 2011) and sequences shorter than 18 nt were discarded. Processed data were analyzed using miRDeep2 core algorithm to identify any new miRNAs and also to assess the authentic mature miRNA sequences (Friedländer et al. 2011). *Nematostella* genome from the NCBI database was used as a reference, and the mature miRNA and miRNA precursor were retrieved from miRBase Release 21 (Kozomara and Griffiths-Jones 2014). The small RNA sequencing data of *Hydra magnipapillata* and *Amphimedon queenslandica* were retrieved from data deposited at NCBI Sequence Read Archive (SRA050926) (Krishna et al. 2012) and (SRP000624) (Grimson et al. 2008), respectively. For miRNA quantification the miRDeep2 quantification algorithm was used and the spike-ins were used for normalizing the read counts. To determine the miRNA isoforms, the data were analyzed using miRDeep2 and the different length miRNA isoforms were identified from the files produced by miRDeep2 for individual miRNAs. To analyze both multiple isoforms and nontemplate nucleotide additions, we considered the guide miRNAs with read count above 50 as cut-off. The heat maps were generated using MultiExperiment Viewer (MeV version 4.7) (http://mev.tm4.org/; last accessed May 4, 2017).

## Results

### miRNAs in Cnidaria and Porifera Exhibit Multiple Isoforms

**Fig. 1.**
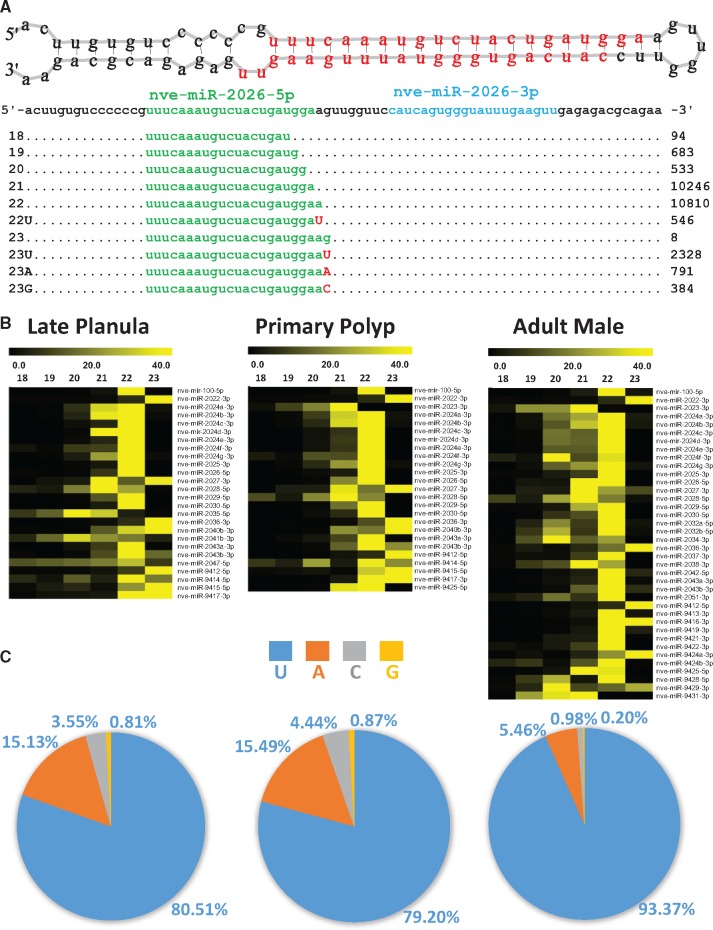
—Posttranscriptional modifications of miRNAs in the cnidarian *Nematostella vectensis* during late planula, primary polyp, and adult male development stages. (*A*) Small RNA reads mapped on nve-miR-2026 hairpin. The miRNA reads with 3′ modifications including isoforms and 3′ nontemplated additions are aligned below, with the read counts shown on right, and the designated miRNA, miRNA*, and nontemplated nucleotide species colored green, blue, and red, respectively. (*B*) Heat map of miRNA isoforms expression across late planula, primary polyp and adult male developmental stages. Fractions of different sizes of miRNA reads mapped to each premiRNA sequence, each line denotes one miRNA. (*C*) Frequency of modified 3′ nontemplated miRNAs at 23rd nt position observed across late planula, primary polyp, and adult male development stages. The pie chart presents percentage sum of variant reads with each nontemplated nucleotide (U, A, C, and G) modifications added at 23rd nt of miRNAs 3′ end.

**Fig. 2.**
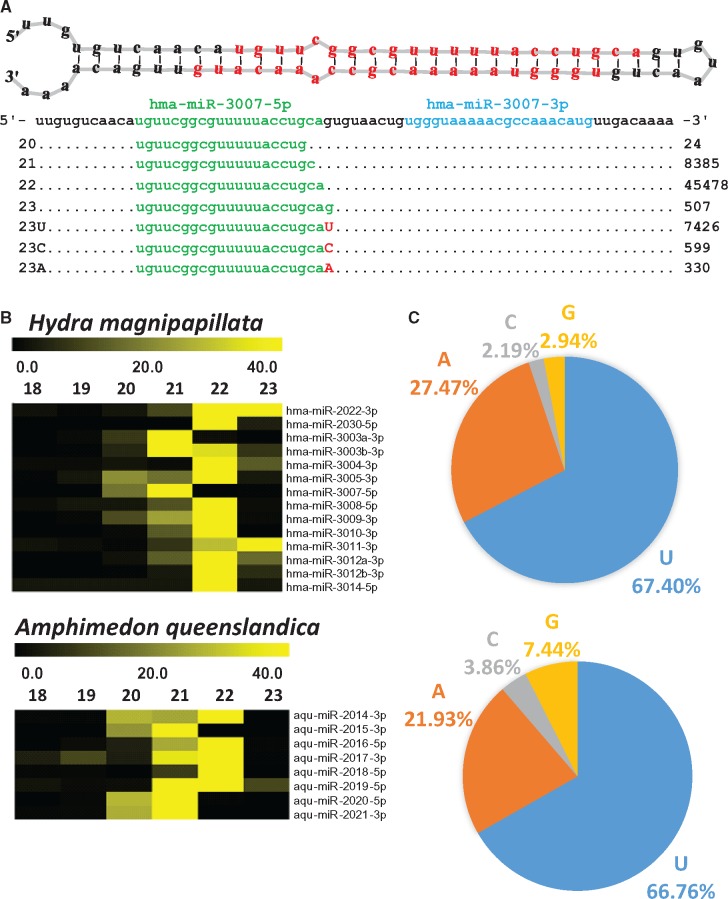
—Posttranscriptional modifications of miRNAs in the cnidarian *Hydra magnipapillata* and poriferan *Amphimedon queenslandica*. (*A*) Small RNA reads mapped on hma-miR-3007 hairpin. The miRNA reads with 3′ modifications including isoforms and nontemplated additions are aligned below, with the read counts shown on right, and the designated miRNA, miRNA*, and nontemplated nucleotide species colored green, blue, and red, respectively. (*B*) Heat map of miRNA isoforms expression analyzed from *Hydra magnipapillata* and *Amphimedon queenslandica* small RNA sequinning data. Fractions of variant isoforms of miRNA reads mapped to each premiRNA sequence, each line denotes one miRNA. (*C*) Frequency of modified 3′ nontemplated miRNAs at 23rd nt position observed from *H. magnipapillata* and *A. queenslandica* small RNA sequinning data. The pie chart presents percentage sum of variant reads with each nontemplated nucleotide (U, A, C, and G) modification added at 23rd nt of miRNAs 3′ end.

In order to identify multiple isoforms expressed with varied length in nonbilaterian animals, we analyzed the small RNA sequence data from *Nematostella vectensis, Amphimedon queenslandica*, and *Hydra magnipapillata*. In *Nematostella* we analyzed small novel RNA sequencing data we generated from distinct RNA preparations of late planula, primary polyp and adult male. The small RNA sequencing data of *Hydra* and *Amphimedon* were retrieved from previously published data (Grimson et al. 2008; Krishna et al. 2012). To identify the miRNA isoforms with different length, we analyzed the output generated by miRDeep2 (Friedländer et al. 2011) analysis and read counts of each miRNA matching 18–24 nt sequence length without any mismatches were collected (supplementary table 4, Supplementary Material online). We observed that all the three species expressed miRNAs with variable length isoforms (figs. 1*A* and 2*A*), the data was plotted on heatmaps (figs. 1*B* and 2*B*). When comparing the percentage of total reads distributed among different lengths, we observed that the majority of reads distributed among 21 and 22 nt length miRNAs. Yet, a substantial fraction of the reads were of different lengths. In case of *N. vectensis* we found that miRNA length variants are consistent throughout different development stages (fig. 1*B*). In overall, our analysis reveals that in nonbilaterian animals miRNAs exhibit heterogeneity in length like in bilaterian animals.

### miRNAs of Cnidaria and Porifera Carry Nontemplated Addition at Their 3′ End

In order to identify the nontemplate modification at 3′ ends of miRNAs, we analyzed the miRNA sequence data from *Amphimedon*, *Hydra*, and *Nematostella* using miRDeep2 and two mismatches were permitted. The miRNA sequences with single U or A mismatches to the genome at the 3′ end were considered as miRNAs carrying nontemplated additions. Initial analysis was performed to detect the 3′ nontemplate modifications at positions 21–24 of miRNAs (supplementary table 4, Supplementary Material online). However, in further analysis we focused on 23 nt-long miRNAs, as we observed that the 23rd nt is the one that undergoes nontemplate modifications most frequently. The read counts from miRNAs with 3′ nontemplated modifications were normalized and the percentage of each nucleotide addition was calculated. We observed that mono-uridylation was the most frequent modification and mono-adenylation is the second most frequent nontemplate modification (figs. 1*C* and 2*C*). To exclude the possibility that the observed heterogeneous length isoforms and 3′ nontemplated modifications are caused by library generation or sequencing errors, we examined the small RNA sequencing data generated by different small RNA sequencing studies of *Nematostella vectensis* (SRA accession number SRP000409) (Moran et al. 2014) and *Hydra magnipapillata* (SRA accession number SRP037736) (Lim, Anand, et al. 2014). We found that these miRNA variations were similar in all the datasets analyzed (supplementary table 4, Supplementary Material online). Additionally, we also performed relative analysis on mismatches at the internal positions of miRNAs. The 3' terminal nontemplate additions at 23rd position were found to have relatively 60–80 fold higher read counts than any other base modification at internal positions of mature miRNAs (supplementary table 5, Supplementary Material online), further supporting the notion that the 3′ terminal nontemplate additions are genuine and are not the result of a technical artefact. Overall, our analysis reveals that in nonbilaterian animal miRNAs undergo 3′ nontemplated modifications similarly to bilaterian animals.

### Phylogenetic Analysis of Metazoan TUTases

From an initial comparative analysis on TUTases in both *Drosophila* and humans we have noticed changes in protein domain assembly. Thus, we decided to perform classification of all TUTases in human, zebrafish and *Drosophila* through phylogenetic analysis to understand the relationship of TUTases among these three species.

**Fig. 3.**
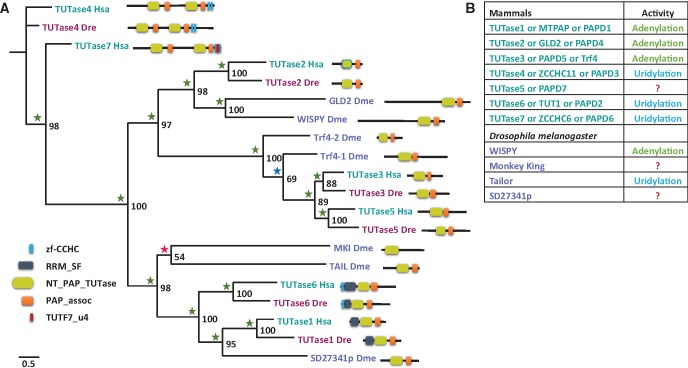
—A phylogenetic relationship of TUTases from Human, *Drosophila melanogaster* and zebrafish. (*A*) A PhyML tree was constructed with VT model (+I + G). Bootstrap support values above 50% are indicated above branches. Values from Bayesian tree are indicated by a green (PP = 1.0), blue (0.95 ≤ PP < 1.0), or red (0.7 < PP < 0.95) star. (*B*) TUTases with known activity from mammals and *Drosophila melanogaster*. Abbreviations of species names are: Dre, *Danio rerio*; Dme, *Drosophila melanogaster*; Hsa, *Homo sapiens* (human).

Through similarity search we retrieved the protein sequences from all the three species and the domain architecture of retrieved proteins were analyzed. Our analysis showed that TUTase2 was duplicated in *Drosophila* into GLD2 and Wispy. In addition, we noted that TUTases6 duplication in the *Drosophila* lineage resulted in Tailor and Monkey King (fig. 3). Apart from TUTase2 and TUTase6 protein duplications in *Drosophila*, we also found changes in domain composition of TUTases between human and *Drosophila*. Noticeably, neither Tailor nor Monkey King have the RNA binding domain present in its ancestor, TUTase6 (fig. 3). We were not able to detect the TUTase7 in both zebrafish and *Drosophila*. In *Drosophila* along with TUTase7, TUTase4 was also absent, suggesting a loss. A more detailed analysis of the phylogeny TUTases 4 and 7 is discussed below.

In overall, our phylogenetic analysis of TUTases from all three species, showed that in *Drosophila* TUTases have undergone major diversification, such as duplication, loss of protein and changes in domain composition. To gain further in-depth understanding of TUTases evolution, we performed phylogenetic analyses of each individual TUTase family.

### Phylogenetic Analysis of the TUTases 4, 7, and 2 Family

**Fig. 4.**
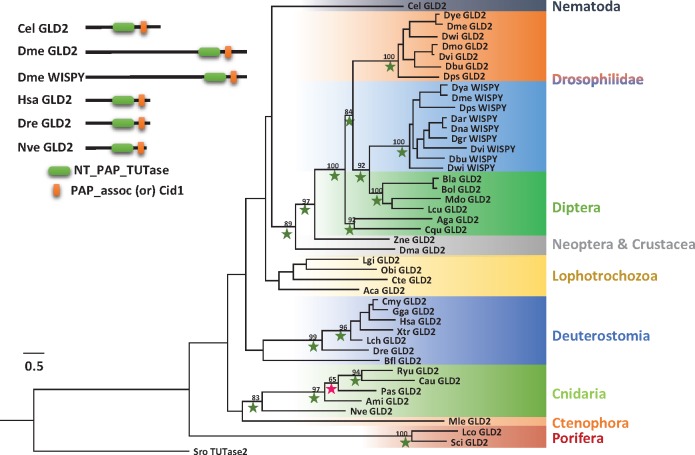
—A phylogenetic tree of GLD2 and Wispy of selected animals. Schematic representation of GLD2 and Wispy protein domain composition from several species. A PhyML tree was constructed with JTT model (+I + G). Bootstrap support values above 50% are indicated above branches. Values from Bayesian tree are indicated by a green (PP = 1.0), blue (0.95 ≤ PP < 1.0), or red (0.7 < PP < 0.95) star. Abbreviations of species names are: Ami, *Acropora millepora*; Aca, *Aplysia californica*; Aga, *Anopheles gambiae*; Bfl, *Branchiostoma floridae*; Bol, *Bactrocera oleae*; Bla, *Bactrocera latifrons*; Cau, *Corynactis australis*; Cmy, *Chelonia mydas*; Cel, *Caenorhabditis elegans*; Cte, *Capitella teleta*; Cgi, *Crassostrea gigas*; Cqu, *Culex quinquefasciatus*; Dre, *Danio rerio*; Dma, *Daphnia magna*; Dbu, *Drosophila busckii*; Dgr, *Drosophila grimshawi*; Dya, *Drosophila yakuba*; Dan*, Drosophila ananassae*; Dsi, *Drosophila simulans*; Der, *Drosophila erecta*; Dps, *Drosophila pseudoobscura*; Dwi, *Drosophila willistoni*; Dvi, *Drosophila virilis*; Gga, *Gallus gallus*; Hsa, *Homo sapiens*; Lch*, Latimeria chalumnae*; Lgi, *Lottia gigantean*; Lcu, *Lucilia cuprina*; Lco, *Leucosolenia complicata*; Mle, *Mnemiopsis leidyi*; Mdo, *Musca domestica*; Nve, *Nematostella vectensis*; Obi, *Octopus bimaculoides*; Pas, *Porites astreoides*; Ryu, *Ricordea Yuma*; Sro, *Salpingoeca rosetta*; Sci, *Sycon ciliatum*; Xtr, *Xenopus tropicalis;* Zne, *Zootermopsis nevadensis*.

To gain deeper insight on evolution of TUTase2, we constructed a phylogenetic tree of TUTase2 (GLD2) and Wispy from most major phyla of animals and other holozoans. Initially we did not find exact matches for TUTase2 in Choanoflagellata or *Capsaspora*, however, there were partial sequence matches. These partially matching proteins were mostly excluded from our phylogeny due to their limited homology, but we used the homolog from the choanoflagellate *Salpingoeca rosetta* as an outgroup. Among Porifera, we were not able to find the TUTase2 in class Demospongiae (*Stylissa carteri, Xestospongia testudinaria, Haliclona tubifera*, and *A. queenslandica*). This is probably the result of a loss of TUTase2 that is specific to demosponges as we were able to find TUTase2 homologs in the Calcarea and Homoscleromorpha sponge classes (fig. 4). However, due to the lack of publicly available assembled transcriptome data from the Hexactinellida we could not determine whether this sponge class carries TUTase2. From our initial phylogenetic analysis, we know that TUTase2 was duplicated in *Drosophila* into TUTase2 (GLD2) and Wispy (fig. 3), in order to find at what evolutionary time point the duplication of TUTase2 took place we performed a detailed sequence survey in all available classes of Hexapoda. Interestingly, we found both GLD2 and Wispy only in the Drosophilidae family. Even other insect families within Diptera such as Culicidae, Calliphoridae, Schizophora, and Bactrocera, which are the closest relatives of Drosophilidae, lack duplication of TUTase2. Hence, it appears that the TUTase2 duplication occurred in the last common ancestor of Drosophilidae (fig. 3). Further, in terms of domain organization, TUTase2 consists of NT_PAP_TUTase and PAP_assoc or Cid1 domain at the C-terminal region of the protein (Wang et al. 2002). In our analysis, we observed that both domains were conserved throughout metazoan TUTase2. In Drosophilidae, GLD2 and Wispy have both domains, but there is a unique extension of the N-terminal region in both proteins, further supporting the notion that this duplication is Drosophilidae specific (fig. 4).

**Fig. 5.**
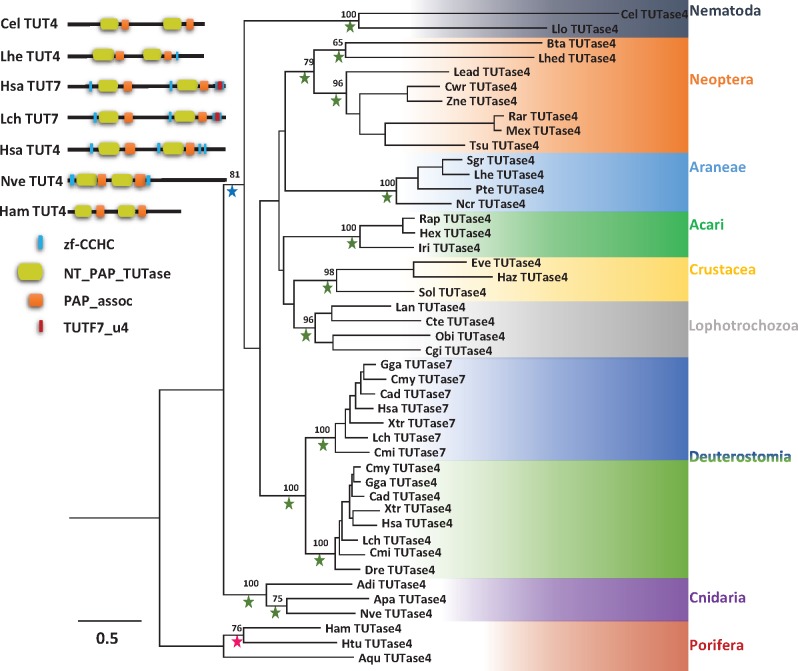
—A phylogenetic tree of TUTase4 and TUTase7 of selected animals. Schematic representation of TUTase4 and TUTase7 protein domain composition from several species. A PhyML tree was constructed with LG model (+I + G). Bootstrap support values above 50% are indicated above branches. Values from Bayesian tree are indicated by a green (PP = 1.0), blue (0.95 ≤ PP < 1.0), or red (0.7 < PP < 0.95) star. Abbreviations of species names are: Aqu, *Amphimedon queenslandica*; Apa, *Aiptasia pallida*; Adi, *Acropora digitifera*; Bfl, *Branchiostoma floridae*; Bta, *Bemisia tabaci*; Cmi, *Callorhinchus milii*; Cad, *Crotalus adamanteus*; Cel, *Caenorhabditis elegans*; Cte, *Capitella teleta*; Cgi, *Crassostrea gigas*; Cwr, *Cryptocercus wright*; Dre, *Danio rerio*; Dma, *Daphnia magna*; Eve, *Eulimnogammarus verrucosus*; Gga, *Gallus gallus*; Haz, *Hyalella Azteca*; Hex, *Hyalomma excavatum*; Htu, *Haliclona tubifera*; Ham, *Haliclona amboinensis*; Hsa, *Homo sapiens*; Iri, *Ixodes ricinus*; Lch, *Latimeria chalumnae*; Llo, *Loa loa*; Obi, *Octopus bimaculoides*; Lan, *Lingula anatine*; Lhe, *Latrodectus Hesperus*; Lhe, *Lygus Hesperus*; Lsp, *Leuctra sp. AD-2013;* Mex, *Medauroidea extradentata*; Ncr, *Nephilengys cruentata*; Nve, *Nematostella vectensis*; Pte, *Parasteatoda tepidariorum*; Rar, *Ramulus artemis*; Rap, *Rhipicephalus appendiculatus*; Sgr, *Steatoda grossa*; Sol, *Scylla olivacea*; Tsu, *Tanzaniophasma subsolana*; Xtr, *Xenopus tropicalis*; Zne, *Zootermopsis nevadensis*.

Apart from mono adenylation many miRNAs are modified by mono uridylation at their 3′ end. As mentioned earlier TUTase4 and TUTase7 were found to be responsible for 3′ terminal mono-uridylation in mammalian models. However, their evolutionary position in the broader Metazoa kingdom is unclear. We retrieved by homology search the TUTase4 in all major phyla of Metazoa (supplementary table 1, Supplementary Material online), except the order Diptera where we noticed its absence. In the case of nonanimal holozoans TUTase4 was absent from both Choanoflagellata and *Capsaspora*. As we observed that TUTase4 is absent in Diptera we continued searching in other Classes of Hexapoda. Unlike Diptera, other subphyla of arthropods, including the other groups of Neoptera (winged insects) do carry TUTase4 in their genome. This strongly suggests a dipteran-specific loss of TUTase4 (fig. 5).

Unlike TUTase4 orthologs that could be found in the vast majority of metazoans, including poriferans and cnidarians (fig. 5), TUTase7 is found only in Tetrapods as well as the Coelacanth *Latimeria chalumnae* and the Australian ghostshark *Callorhinchus milii*. Based on our analysis TUTase7 has evolved in vertebrates and it is a duplication of TUTase4 that gained an additional new domain, TUTF7_u4 (fig. 5).

### Phylogenetic Analysis of Tailor, Monkey King, TUTase6 and TUTase1

**Fig. 6.**
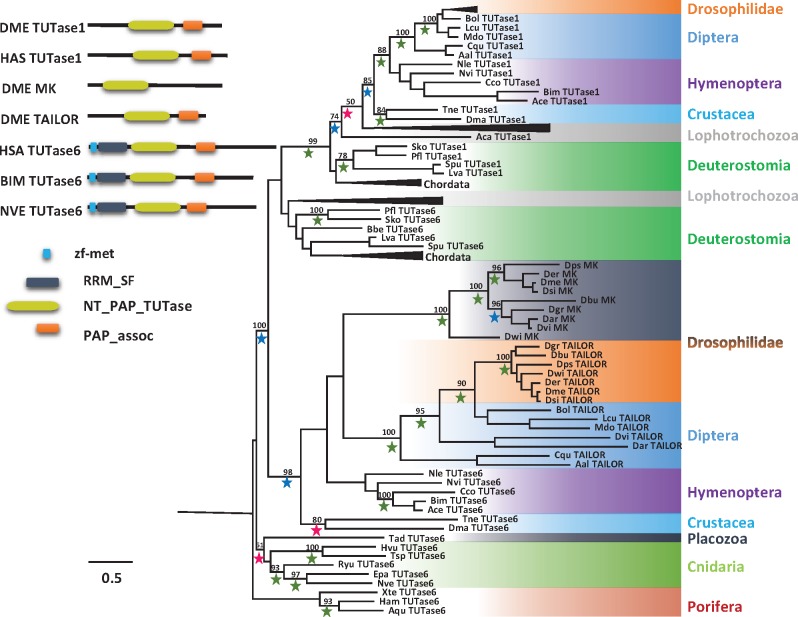
—A phylogenetic tree of TUTase1, TUTase6, Tailor, and Monkey king of selected animals. Schematic representation of TUTase1, TUTase6, Tailor, and Monkey king protein domain composition from several species. A PhyML tree was constructed with LG model (+I +G). Bootstrap support values above 50% are indicated above branches. Values from Bayesian tree are indicated by a green (PP = 1.0), blue (0.95 ≤ PP < 1.0), or red (0.7 < PP < 0.95) star. Abbreviations of species names are: Aqu, *Amphimedon queenslandica*; Apa, *Aiptasia pallida*; Ace, *Apis cerana*; Aal, *Aedes albopictus*; Aca, *Aplysia californica*; Bbe, *Branchiostoma belcheri*; Bim*, Bombus impatiens*; Bol, *Bactrocera olea*e; Cte, *Capitella teleta*; Cco, *Cyphomyrmex costatus*; Cqu, *Culex quinquefasciatus*; Che, *Clytia hemisphaerica*; Dre, *Danio rerio*; Dma, *Daphnia magna*; Dbu, *Drosophila busckii*; Dgr, *Drosophila grimshawi*; Dsi, *Drosophila simulans*; Der, *Drosophila erecta*; Dps, *Drosophila pseudoobscura*; Dwi, *Drosophila willistoni*; Dvi, *Drosophila virilis*; Dar, *Drosophila arizonae*; Dme*, Drosophila melanogaster*; Ham, *Haliclona amboinensis*; Hvu, *Hydra vulgaris*; Hsa, *Homo sapiens*; Lva, *Lytechinus variegatus*; Lan, *Lingula anatine*; Lpo, *Limulus Polyphemus*; Lcu, *Lucilia cuprina*; Mdo*, Musca domestica*; Nvi*, Nasonia vitripennis*; Nle, *Neodiprion lecontei*; Nve, *Nematostella vectensis*; Pfl, *Ptychodera flava*; Ryu, *Ricordea Yuma*; Sko, *Saccoglossus kowalevskii*; Sca, *Stylissa carteri*; Spu, *Strongylocentrotus purpuratus*; Tsp*, Turritopsis sp. SK-2016*; Tne, *Triops newberryi*; Obi, *Octopus bimaculoides*; Xte, *Xestospongia testudinaria*; Xtr, *Xenopus tropicalis*.

We noticed in our initial phylogenetic analysis (fig. 2) that Tailor and Monkey King of *Drosophila* could originate from a TUTase6 duplication. When we performed sequence similarity searches for TUTase6 and TUTase1 in all the major groups of animals. We found that TUTase6 is conserved throughout Metazoa. In order to find at what time point the TUTase6 was duplicated into Tailor and Monkey King, we performed detail sequence search in all available classes of Hexapoda. Interestingly, we found the duplication of TUTase6 is unique to Drosophilidae family of insects, which is similar to what we detected from our previous analysis on TUTase2 duplication is Drosophilidae specific. In terms of domain architecture Tailor and Monkey King have lost RNA binding domain found in its ancestor protein TUTase6. In addition, Monkey King also lost its Poly(A) polymerases (PAPs) associated domain (fig. 6).

In contrast, to TUTase6 which is conserved across Metazoa including all the nonbilaterian animals as well as in Choanoflagellata and *Capsaspora* (supplementary table 1, Supplementary Material online), TUTase1 (also known as Mitochondrial Poly(A) Polymerase, MTPAP) was absent from all nonbilaterian animals and other nonmetazoan holozoans including Choanoflagellata, *Capsaspora*, Porifera, Ctenophora, Placozoa, and Cnidaria (fig. 6). Based on our phylogenetic analysis, TUTase1 probably has been derived from a gene duplication of TUTase6 in the last common ancestor of bilaterian animals, the Urbilateria.

## Discussion

The study of miRNAs in nonbilaterian models has opened new perceptive on evolution of miRNA as an ancestral RNA regulatory mechanism (Grimson et al. 2008; Moran et al. 2013). To test the possibility that miRNAs in nonbilaterian animals are processed into multiple isoforms like in bilaterians, we analyzed the small RNA sequencing data from two cnidarians (*Nematostella vectensis and Hydra magnipapillata*) and one poriferan (*Amphimedon queenslandica*). Our analysis revealed that in all three species miRNAs frequently undergo modifications like in bilaterians: the majority of miRNAs are expressed as different length isoforms and frequent modifications of the 3′ end by mono U or mono A tailing are observed (figs. 1 and 2). However, the functional consequences of expressing multiple isoforms in these three species is unknown. We believe that this modification may be part of the tailing and trimming mechanism, which includes adding or removing nucleotides at 3′ ends of small RNAs after they are loaded into AGO (Ameres et al. 2010).

**Fig. 7.**
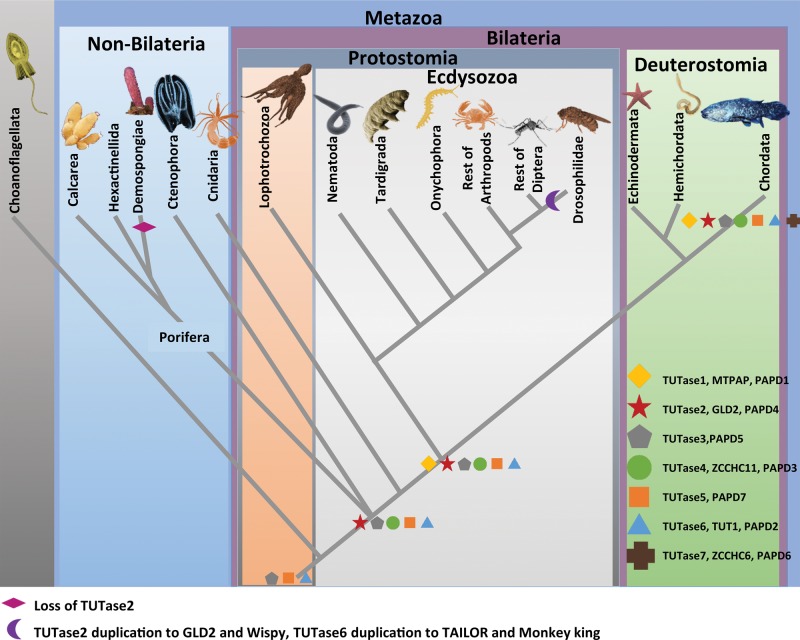
—Origin and evolution of RNA modifying enzymes in Metazoa. A schematic tree of major animal groups with loss and gain events marked on relevant branches.

Based on our analyses of miRNA 3′ nontemplated modifications in nonbilaterian animals, we observed that mono-uridylation is the most frequent modification (figs. 1*C* and 2*C*). As mentioned earlier, these 3′ nontemplated nucleotide modifications were shown to be mediated by TUTases in bilaterian animals (Kwak and Wickens 2007; Norbury 2013; Thornton et al. 2014; Kim et al. 2015). Among all TUTases, TUTase4 (ZCCHC11) and TUTase7 (ZCCHC6) are known to regulate miRNA 3′ uridylation (Heo et al. 2012; Lim, Ha, et al. 2014; Thornton et al. 2014; Kim et al. 2015). Yet, their evolutionary history in the broader Metazoa kingdom was unclear. Based on our analysis TUTase4 is a metazoan innovation, as TUTase4 homologs are absent from nonanimal holozoans including Choanoflagellata and *Capsaspora* (fig. 7). Another curious finding is a dipteran-specific loss of TUTase4 (fig. 5). Despite the fact that Dipterans like *Drosophila* have lost the TUTase4, they still possess a small RNA 3′ uridylation mechanism and it was shown that the *Drosophila*-specific TUTase Tailor is responsible for it (Bortolamiol-Becet et al. 2015; Reimao-Pinto et al. 2015). Taking this into consideration, our phylogenetic analysis reveals that in the *Drosophila* lineage a TUTase6-related protein (Tailor) took over the roles of TUTase4 that was lost.

In contrast to TUTase4, TUTase7 is found only in subgroups of vertebrates (figs. 5 and 7) and it is most probably a duplication of TUTase4 that gained an additional new domain, TUTF7_u4 (fig. 5). Additionally, lack of this unique domain in TUTase4 also supports the notion that TUTase4 has evolved earlier than TUTase7. However, the function of this newly attained domain is unknown. Interestingly, the phyletic distribution of TUTase7 suggests that it was lost specifically in teleost fish, which is the largest group of vertebrates, raising again the question whether it carries any unique functions that are not shared with TUTase4.

Modification of certain miRNAs at their 3′ end by addition of mono (A) has been noted in several organisms and TUTase2 was found to responsible for this modification (Ameres et al. 2010; D’Ambrogio et al. 2012; Katoh et al. 2015). Further, knockdown of TUTase2 has shown that 3′ monoadenylation regulates miRNA (mir-122) stability (Katoh et al. 2009). From our analysis on miRNA 3′ nontemplated modifications we noticed that miRNAs from nonbilaterian animals undergo 3′ mono adenylation as well (figs. 1*C* and 2*C*). This suggests that TUTase2 may have a role in regulating miRNA stability in these nonbilaterian animals and that this function might be ancestral. Through homology search, we found TUTase2 in Cnidaria and Porifera. However, we were not able to find TUTase2 homologs in Demospongiae, a class of Porifera. This is probably the result of a loss of TUTase2 that is specific to demosponges as we are able to find TUTase2 homologs in other sponge classes and it suggests that the evolution of TUTases might be dynamic in different nonbilaterian groups (fig. 7).

In the case of Drosophilidae, we found unprecedented number of modifications in TUTase family of proteins (figs. 3 and 7), the TUTase2 duplication to GLD2 and Wispy is one of them. From phylogenetic analysis it appears that the TUTase2 duplication occurred in the last common ancestor of Drosophilidae (fig. 4). In addition, from domain organization analysis we noted that both GLD2 and Wispy have unique extension of N-terminal region (fig. 4), further supporting the lineage-specificity of this duplication is Drosophilidae specific. Despite GLD2 and Wispy sharing common domains their organization is slightly different (fig. 4). Wispy is a Drosophilidae specific homolog of GLD2 known to regulate the poly(A) tail of specific mRNAs during oocyte maturation in *Drosophila* (Cui et al. 2008). Additionally, Wispy is also responsible for miRNA 3′ adenylation and facilities miRNA downregulation (Lee et al. 2014). GLD2 function in *Drosophila* and its possible overlap with Wispy is currently unknown.

Beside the TUTase2 duplication in Drosophilidae, we found that TUTase6 is also duplicated into Tailor and Monkey King specifically in this lineage (fig. 6). TUTase6 was first identified as a stability regulator of U6 snRNA in humans (Trippe et al. 1998). However, recent studies of the TUTase6 role in miRNA expression, showed that it has direct effect on 3′ nucleotide addition to specific miRNAs (Knouf et al. 2013). We noticed in our initial phylogenetic analysis (fig. 3) that Tailor and Monkey King of *Drosophila* could originate from a TUTase6 duplication. As mentioned earlier, the *Drosophila* Tailor is directly involved in catalysis of the miRNA 3′ uridylation. Deletion of Tailor in *Drosophila* S2 cells increases the levels of mirtrons, a class of noncanonical splicing-mediated miRNAs. This demonstrates that Tailor has a role in negatively regulating mirtrons accumulation (Bortolamiol-Becet et al. 2015; Reimao-Pinto et al. 2015). To the best of our knowledge, the function of Monkey King has not been published. Further, In terms of domain architecture Tailor and Monkey King have lost RNA binding domain found in their ancestor TUTase6 and Monkey King also lost its Poly(A) polymerases (PAPs) associated domain (fig. 6). These findings again suggest that there might have been sub or neo-functionalization in Drosophila between Tailor and Monkey King in relation to the ancestral TUTase6. As in previous cases, this duplication of TUTase6 seems to be specific to Drosophilidae (fig. 6). The numerous lineage-specific changes of TUTases in Drosophilidae hint at an accelerated evolutionary rate of this gene family in fruit flies and further suggest that *Drosophila*, while being an extremely important model organism for the functional study of RNA metabolism in animals, probably possess a highly derived RNA tailing mechanism.

In addition to TUTase6 duplication in Drosophilidae, we noted that TUTase1 probably has been derived from a gene duplication of TUTase6 in Urbilateria (fig. 7). Studies have shown the TUTase1 is crucial in regulating stability of mammalian mitochondrial transcripts by adding poly(A) tails (Tomecki et al. 2004; Nagaike et al. 2005). In contrast, mitochondrial mRNA polyadenylation in plants leads to degradation (Schuster and Stern 2009). In yeast, the mitochondrial mRNAs lack poly(A) tails and the stability of mitochondrial mRNA is regulated by mRNA-binding proteins (Dieckmann et al. 1984; Butow et al. 1989). The regulation of mitochondrial transcripts in nonbilaterian animals and whether it involves the activity of any TUTases is currently completely unknown and deserves further study as it can reveal what was the ancestral mechanism of mitochondrial mRNA stabilization in animals.

In overall, our study reveals that three members of the TUTase family, TUTases 3, 5, and 6, were most likely already present in the last common ancestor of *Capsaspora* and Metazoa. Further, other major transitions in the TUTase family that our study reveals include the emergence of TUTase2 and TUTase4 at the base of Metazoa, the loss TUTase2 in demosponges, and the emergence of TUTase1 is bilaterians (fig. 7).

## Conclusions

We found that miRNAs in nonbilaterian animals often undergo modifications by tailing like in bilaterians, suggesting that this RNA stability control system was already present in the last common ancestor of all animals. However, variation in the components of this system exists in some animal lineages, with Drosophilidae being the most striking example for substantial divergence. By phylogenetic studies on TUTases, we revealed several important events in the evolution of this protein family in the animal kingdom (fig. 7). Lastly, we believe that future functional studies of TUTases in miRNA modification of nonbilaterian models will provide new insight into the evolution of small RNA pathways in animals.

## Supplementary Material

Supplementary data are available at *Genome Biology and Evolution* online.

## Supplementary Material

Supplementary Data
